# Mental health in adolescents with Type 1 diabetes: results from a large population-based study

**DOI:** 10.1186/1472-6823-14-83

**Published:** 2014-10-10

**Authors:** Børge Sivertsen, Keith J Petrie, Ane Wilhelmsen-Langeland, Mari Hysing

**Affiliations:** Division of Mental Health, Norwegian Institute of Public Health, Kalfarveien 31, 5018 Bergen, Norway; Uni Research Health, P.O.Box 7810, N-5020 Bergen, Norway; Department of Psychiatry, Helse Fonna HF, P.O.Box 2170, N-5504 Haugesund, Norway; Department of Psychological Medicine, University of Auckland, Auckland, 1142 New Zealand; The Institute for Psychological Counselling, 5012 Bergen, Norway; The Regional Centre for Child and Youth Mental Health and Child Welfare, Uni Research Health, P.O.Box 7810, N-5020 Bergen, Norway

**Keywords:** Type 1 diabetes, Mental health, Eating disturbances, Sleep, Correlates, Epidemiology

## Abstract

**Background:**

Diabetes has previously been linked to mental health problems in adolescents, but more recent studies have yielded mixed findings. The aim of the current study was to compare symptoms of mental health problems, sleep and eating disturbances in adolescents with and without Type 1 diabetes in a population based sample.

**Methods:**

Data were taken from the youth@hordaland study, a large population based study in Hordaland County in Norway conducted in 2012. In all, 9883 adolescents aged 16–19 years (53% girls) provided self-reported data on both diabetes and a range of instruments assessing mental health symptoms, including depression, anxiety, obsessive-compulsive behaviours, hyperactivity, impulsivity, inattention, perfectionism, resilience, sleep problems and eating behaviour.

**Results:**

40 adolescents were classified as having Type 1 diabetes (prevalence 0.4%). We found that adolescents with Type 1 diabetes did not differ from their peers on any of the mental health measures.

**Conclusions:**

This is one of the first population-based studies to examine mental health of adolescents with Type 1 diabetes. There was no evidence of increased psychopathology across a wide range of mental health measures. These findings contradict previous studies, and suggest that Type 1 diabetes is not associated with an increased risk of psychosocial problems.

## Background

Type 1 diabetes in adolescence has previously been linked to a range of psychosocial problems [1–3]. In a review from 2009, Kakleas and colleagues concluded that Type 1 diabetes in adolescents was associated with increased risk of developing psychiatric disorders (10–20%), specifically eating disturbances (8–30%) [4]. Type 1 diabetes in adolescence has also previously been linked to a reduced quality of life [5], increased alcohol use [6], as well as worse sleep [7]. Similarly, a meta-analyses on children concluded that diabetic children were more likely to experience various psychological difficulties compared to their healthy peers [8]. However, these effects were small in magnitude, and also weaker in the more recent studies and studies with well-matched comparison groups [8]. Furthermore, a large and well-controlled study of 2672 adolescents (aged 10–21) with diabetes found no differences in depressive symptoms between adolescents with or without diabetes [9].

Still, the vast majority of studies of mental health in adolescents with Type 1 diabetes has been in small clinical studies and there is a strong need for population-based studies to compare mental health comorbidities among adolescents with and without Type 1 diabetes.

Technological and medical advances in therapy have changed the everyday life of adolescents with Type 1 diabetes dramatically the last decade. Now 64% of Norwegian children and adolescents use insulin pump therapy [10]. There are some indications that recent improvements may also be reflected in improved mental health [4]. Improved metabolic control may also limit the associated cognitive and emotional problems, and may be one pathway to improved well-being, by reducing the daily hassles of the traditional complex diabetes management. Yet, the management of Type 1 diabetes is still psychologically complex, as improved technology does not change the challenge of psychologically dealing with choices such as insulin dosage and cognitive and emotional reactions to high and low blood sugar levels. But overall, life with Type 1 diabetes has changed dramatically over the last decade, and conclusions regarding mental health, sleep and life-style may need to be reconsidered.

Based on these considerations, the aim of the current study was to compare symptoms of mental health problems, sleep and eating disturbances in adolescents with and without Type 1 diabetes in a population based sample. Data for this study are drawn from a large population-based survey conducted in 2012 of 9883 Norwegian adolescents aged 16–19, (the youth@hordaland study).

## Methods

### Design and procedure

In this population-based study, we used data from the youth@hordaland study of adolescents in the county of Hordaland in Western Norway. All adolescent born between 1993 and 1995 and all students attending secondary education during spring 2012 were invited to participate. Data were collected during spring 2012. Adolescents in secondary education received information about the study by e-mail, and time during regular school hours was allocated to complete the questionnaire. Those not in school received information by post to their home addresses. Also, regional hospitals were contacted so that adolescents that were inpatients at the time of the survey had the opportunity to participate.

### Ethics

The study was approved by the Regional Committee for Medical and Health Research Ethics (REC) in Western Norway. In accordance with the regulations from the REC and Norwegian health authorities, adolescents aged 16 years and older can make decisions regarding their own health (including participation in health studies), and thus gave consent themselves to participate in the current study. Parents/guardians have the right to be informed, and in the current study, all parents/guardians received written information about the study in advance.

### Sample

A total of 19,439 adolescents were invited to participate in the survey, of whom 10,254 agreed. Of this number, 371 adolescents were omitted due to missing data on the instruments relevant for the current study. This yielded a total sample size of 9,883, representing a participation rate of 51%.

### Instruments

#### Demographical characteristics

Maternal and paternal education were reported separately with three response options; “primary school”, “secondary school”, “college or university”. Participants were also asked whether their parents live together, and a rating of the family’s financial status was assessed by asking how their family economy is compared to most others (1 = “like most others”; 2=”better financial circumstances”, and 3=”poorer financial circumstances”.

#### Diabetes

All adolescents indicated if he/she had diabetes from a checklist that included “diabetes”, “asthma”, and “other chronic illness”. As such, this item did not specifically differentiate between Type 1 and Type 2 diabetes. Given a positive response on this item, the adolescents answered an open-ended question were they provided the name of all medications they were taking. These were coded according to the Anatomical Therapeutic Chemical (ATC) classification system and all mediations in the ATC–subgroup A10A (insulins and analogues) were classified as treatments of Type 1 diabetes, and taken as verification of the positive response on the initial check list. In addition, if the adolescents indicated having another chronic illness on the item described above, they completed an open-ended question where they provided the name of that illness. In all, 828 adolescents responded positively to this item, with the most frequently reported illnesses being atopical dermatitis, neurological, and musculoskeletal conditions.

#### Lifestyle behaviours

Body-mass index (BMI) was calculated from self-reported body weight and height. Physical activity was assessed using one item, derived from the WHO “Health behaviour in school-aged children study” (19): “During the last 7 days, how many days have you been physically active for a minimum of 60 minutes)?” with response ranging from “0” to “7” days. Problematic use of alcohol and drugs was assessed using the CRAFFT-questionnaire, a brief screening tool designed specifically to identify adolescent problematic alcohol and drug use in a medical care setting [11].

#### Depression

Depression was assessed using the short version of the Mood and Feelings Questionnaire (SMFQ) [12]. The SMFQ comprises 13 items assessing depressive symptoms rated on a 3-point Likert scale. A recent study from the youth@hordaland study [13] yielded good psychometric properties of the official Norwegian translation and supported the uni-dimensional structure as described in the original version. The Cronbach’s alpha of the SMFQ in the current study was 0.91.

#### Anxiety

Symptoms of anxiety were identified using the short form five-item version of the SCARED inventory for anxiety disorders [14]. The short-form of the SCARED has showed similar psychometric properties to the full version. No validation studies have been conducted on the Norwegian translation. The Cronbach’s alpha of the SCARED in the current study was 0.68.

#### ADHD symptoms

Symptoms of inattention and hyperactivity were measured using subscales from the official Norwegian translation of the Adult ADHD Self-report Scale (ASRS) [15]. The questionnaire was originally constructed for use in adults, but has recently been validated in adolescents [16]. ASRS is an 18 item self-report scale, comprising 9 items on a hyperactivity-impulsivity subscale and 9 items on an inattention-subscale. A previous validation study has found an inconsistency-adjusted sensitivity of 1.0, a specificity of 0.71, a positive predictive value of 0.52, and a negative predictive value of 1.0 [17]. The Cronbach’s alpha of the ASRS in the current study was 0.89.

#### Obsessive-compulsive behaviour

Obsessive-compulsive behaviour was assessed by the following five questions covering key aspects of obsessive compulsive disorder, as outlined by Thompson [18]: “I wash myself more than normal. I am afraid of infection”, “I often have to check or control things”, “I am concerned with order and symmetry”, “I must often have repeated assurances and answers to questions”, “I have distressing or disturbing thoughts”. These 5 items were rated on a three point Likert scale with response options; “not true”, “somewhat true” and “certainly true”. The Cronbach’s alpha in the current study was 0.71.

#### Resilience

Resilience was assessed by the Resilience Scale for Adolescents (READ) [19], which consists of 28 items rated on a 5-point Likert scale comprising: Personal Competence, Social Competence, Structured Style, Family Cohesion and Social Resources. The READ has shown adequate psychometric properties [20]. The Chronbach’s alpha of the READ in the current study was 0.95.

#### Eating disturbances

Eating disturbances was assessed by the Eating Disturbance Scale (EDS-5) [21], a brief screening instrument for problematic eating in normal populations. The EDS-5 has been shown to have good concurrent and construct validity, and a sensitivity and specificity of 0.90 and 0.88 with respect to DSM-IV eating disorders [21]. The Cronbach’s alpha of the EDS-5 in the current study was 0.75.

#### Perfectionism

Perfectionism was assessed by the short version of the Perfectionism subscale from the Eating Disorder Inventory (EDI) [22]. The scale was adapted to a three points response scale from the original 6-point scale for this study. The Chronbach’s alpha of the EDI in the current study was 0.73.

#### Sleep variables

Self-reported bedtime and rise time were indicated in hours and minutes and were reported separately for weekends and weekdays. Time in bed (TIB) was calculated by subtracting bedtime from rise time. Sleep onset latency (SOL) and wake after sleep onset (WASO) were indicated in hours and minutes, and sleep duration was defined as TIB minus (SOL + WASO). Sleep efficiency was calculated as sleep duration divided by TIB multiplied by 100 (reported as percentage). Subjective sleep need was reported in hours and minutes, and sleep deficiency was calculated separately for weekends and weekdays, subtracting total sleep duration from subjective sleep need.

Insomnia was operationalized according to the DSM-V criteria for insomnia: self-reported difficulties initiating and maintaining sleep for at least three times a week, with a duration of three months or more, as well as tiredness or sleepiness on at least three days per week.

A variable assessing symptoms of obstructive sleep apnoea was also created based on the following two items: 1) “I snore (or someone else says I snore)” (“true” or “partly true”), and 2) reports of “sleepiness” at least three days per week.

#### Subjective health complaints

Subjective health complaints were measured using five items from the thoroughly validated HBSC (Health Behaviour in School-aged Children) symptom checklist [23]. Participants reported the experienced frequency of headache, abdominal pain, back pain, dizziness and pain in neck/shoulders experienced during the last six months on a five-point scale ranging from “more or less every day” to “seldom or never”.

### Statistics

IBM SPSS Statistics 21 for Mac (SPSS Inc., Chicago, Ill) was used for all analyses. Pearson’s chi-square tests and Mann–Whitney U-tests were used to examine differences in demographical, clinical and sleep variables in adolescents with and without diabetes. Between-group effect sizes (pooled SD) were calculated using the Cohen’s *d* formula. As the control group also comprised adolescents with chronic other illnesses (n = 828), all statistical analyses were repeated omitting these individuals form the control group. This, however, did not change the results or conclusions in any way, and therefore the control group used in current study includes all adolescents *not* reporting diabetes.

## Results

### Demographical and clinical characteristics

The mean age of the sample was 17.9 years (range 16–19), and included more girls (53.3%) than boys (46.7%). The vast majority (98%) were high school students. The prevalence rate of Type 1 diabetes was 0.4% in both boys (n = 20) and girls (n = 20). Table 1 shows that there were no significant differences between adolescents with and without Type 1 diabetes in terms of maternal or paternal education, and neither family financial nor co-habitant status differed significantly between the two groups.

**Table 1:** **Demographical characteristics and lifestyle behaviours in adolescents with and without Type 1 diabetes in the youth@hordaland study (n = 9,883)**

	No diabetes (n = 9,843)		Type 1 diabetes (n = 40)		
	%/median	(SD)	%/median	(SD)	***P***-value*
**Demographical characteristics**					
Age, mean	19.9	0.7	19.9	0.8	.48
Gender					.75
Girls, %	53.3%		50.0%		
Boys, %	46.7%		50.0%		
Vocational situation					.64
In high school	97.8%		100%		
Trainee	1.5%		0%		
Not in high school	0.8%		0%		
Maternal education, %					.49
University/college	48.7%		50.6%		
High school	41.3%		43.8%		
Primary school	10.1%		15.6%		
Paternal education, %					.84
University/college	43.1%		38.7%		
High school	46.4%		51.6%		
Primary school	10.6%		9.7%		
Parents live together, %					
No	32.7%		25.6%		.40
Family economy					.28
Approx. like most others	67.4%		57.5%		
Better economy	25.5%		30.0%		
Poorer economy	7.1%		12.5%		
**Lifestyle behaviours**					
Current smoker	13.2%		15.0%		.64
CRAFFT sum score	0.8	(1.2)	0.9	1.3	.54
Physical activity (days/wk), %					.52
None	9.9%		15.4%		
1-3 days	49.7%		46.2%		
4 + days	40.4%		38.5%		
Body-mass index	22.2	3.5	23.2	3.1	.03

*P value is based on Mann–Whitney U tests.

### Type 1 diabetes and lifestyle behaviours

No significant differences were found between the Type 1 diabetes versus non-diabetes group in terms of smoking (*P* = .64) or problematic alcohol or drug use (*P* = .54). The proportion of adolescents with Type 1 diabetes who were physically active 4 days or more per week did not differ from the non-diabetes group (38.5% and 40.4%). However, it should be noted that as many as 15.4% of adolescents with Type 1 diabetes (and 9.9% in the non-diabetes group) did not perform any physical activity during the week, although this difference was not statistical significant (*P* = .52). The mean BMI in the Type 1 diabetes group was significantly higher than in the non-diabetes group (23.2 versus 22.2, *P* = .03).

### Type 1 diabetes and mental health symptoms

Table 2 shows the mean symptom levels across all health instruments in the Type 1 diabetes and non-diabetes groups. In terms of mental health problems, the adolescents with Type 1 diabetes did not report higher levels on *any* of the assessed instruments; including depression, anxiety, obsessive compulsivity, ADHD-symptoms, and perfectionism. The Cohen’s *d* effect sizes were small, ranging from .00 to .16. The same pattern was found for resilience. No significant differences between the Type 1 diabetes and non-diabetes groups were identified for any of the 5 subscales, with small effect sizes ranging from .09 to .27.

**Table 2:** **Health characteristics in adolescents with and without Type 1 diabetes in the youth@hordaland study (n = 9,883)**

	No diabetes (n = 9,843)		Type 1 diabetes (n = 40)			
	%/mean	(SD)	%/mean	(SD)	***P***-value*	Cohen’s ***d***
**Mental health**						
Depression (SMFQ Total score)	5.8	5.8	6.5	5.8	.28	.12
Anxiety (SCARED Total score)	1.5	1.8	1.7	1.8	.53	.11
OCD (OCD Total score)	2.4	2.2	2.4	2.3	.71	.00
Hyperact./imp. (ASRS subscale)	11.8	5.4	12.9	6.1	.40	.19
Inattention (ASRS subscale)	15.0	6.2	15.5	6.5	.99	.08
Perfectionism (EDI Total score)	4.6	2.7	4.3	2.7	.55	.11
Eating disturbances (EDS Total score)	3.2	2.3	3.6	2.8	.62	.16
**Resilience**						
Personal competence	30.1	6.1	28.8	6.5	.22	.21
Personal structure	14.0	3.2	13.7	3.2	.53	.09
Social competence	19.5	4.1	18.4	3.9	.07	.27
Social support	21.5	3.5	20.7	4.4	.31	.20
Family cohesion	2.9	5.1	2.3	5.7	.35	.11
**Sleep**						
Sleep duration	6:25	1:39	6:18	1:37	.63	.07
Sleep efficiency	85.3	17.7	87.0	18.2	.57	.09
Sleep deficiency	2:09	2:30	2:19	2:25	.88	.07
Sleep onset latency	0:47	0:57	0:52	1:22	.69	.07
Wake after sleep onset	0:15	0:39	0:03	0:07	.26	.43
Insomnia (DSM-V)	18.5%		11.1%	.39	.69	
Obstructive sleep apnoea (OSA)	4.1%		6.9%	.34	.16	
**Somatic health**						
HSBC Total score	4.9	4.7	6.5	5.6	.08	.31

*P value is based on Mann–Whitney U tests.

### Type 1 diabetes and eating disturbance

**Figure 1:**
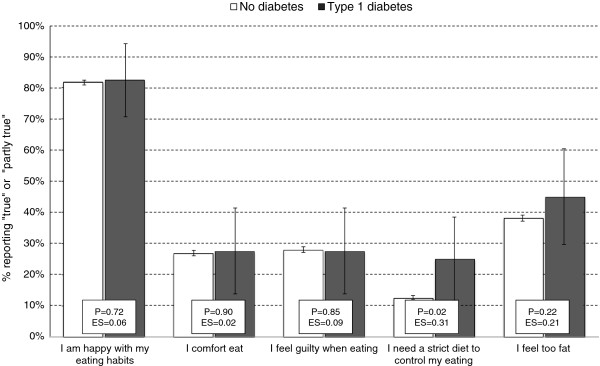
**Eating problems among adolescents with and without Type 1 diabetes in the youth@hordaland study.** Vertical axis represents proportion of adolescents answering “true” or “partly true” on each of the 5 EDS items. Error bars represent 95% confidence intervals. P-values are based on Chi-squared tests, and Cohen’s *d* effect sizes (ES) are calculated from means and standard deviation from the EDS’ original 3 response options.

In terms of eating disturbances, having Type 1 diabetes was not associated with elevated scores on the EDS total score. However, as shown in Figure 1, one of the items in the EDS scale differed between the two groups: a larger proportion of adolescents with Type 1 diabetes reported needing a strict diet to control their eating compared to the non-diabetes group (effect size .31, *P* = .017).

### Type 1 diabetes and sleep problems

Adolescents with Type 1 diabetes did not differ in terms of their reported sleep problems compared to the non-diabetes group on any of the assessed sleep parameters. Neither sleep duration, sleep efficiency, sleep deficiency, SOL nor WASO, differed between the two groups. Similarly, the prevalence of insomnia and OSA was not significantly higher among adolescents with Type 1 diabetes, compared to the control group.

### Type 1 diabetes and subjective health complaints

No significant group differences were found on the HSBC total score, although there was a trend for adolescents with Type 1 diabetes to report more somatic complaints than adolescents without Type 1 diabetes (6.5 versus 4.9, respectively: effect size .31; *P* = .08). Analyses of each of the 5 specific complaints showed that the Type 1 diabetes group reported significantly more back pain (P = .02) than the control group, whereas, no group differences were found for the remaining 4 items (headache, neck and shoulder pain, abdominal pain, and dizziness).

No significant gender differences were found for any of measures.

## Discussion

The aim of this population-based study from was to compare mental health symptoms, sleep problems, health behaviours and eating disturbances in adolescents with and without Type 1 diabetes. We found adolescents with Type 1 diabetes did not differ from adolescents without Type 1 diabetes on any of the instruments used in the current study: Type 1 diabetes was not associated with elevated symptoms of mental health problems, sleep problems, eating disorders nor health behaviours.

The results are in contrast to the elevated level of mental health problems and disturbed eating behaviour found in previous research [4, 24]. While methodological differences may explain some of the discrepancies, it may also reflect recent changes in the management of diabetes. While studies from the 1990s reported that glycaemic control often deteriorated during adolescence [25], the recent rapid adoption of diabetes-pump therapy, especially among children and adolescents, has shown promising results with regards to fewer instances of severe hypoglycaemia, lower HbA1c levels, low risk of ketoacidosis, as well as less glucose fluctuations [26]. It is possible that this improvement in control of diabetes is reflected in mental health, although the current study did not specifically examine the use of insulin pumps. This is supported by some early studies of adolescent and adult pump-users that showed less symptoms of anxiety and depression, as well as improved self-esteem and locus of control after starting therapy [27]. In addition to the direct link between mental health and illness management, psychological functioning may also be affected indirectly through improved cognitive function.

While we have no information on how many of the adolescents with Type 1 diabetes in the current study were using insulin pumps, the adoption rates in Norway are very high, and this may partly explain why so few mental health problems were reported among the adolescents with Type 1 diabetes in the current study. However, use of insulin pump is not likely to be the sole explanation as to why no differences were found across all the assessed domains of psychosocial functioning. Indeed, there are mixed findings with regards to changes in quality of life in persons with diabetes treated with the insulin pump compared with multiple daily injections system [28]. Also, a significant proportion of Norwegian adolescents still use more conventional insulin regimens. As such, there may be other possible factors that may explain why diabetic adolescents in the current study seem to be little affected and restricted by their illness in terms of mental health functioning For example, it is possible that having Type 1 diabetes is now less stigmatizing than before, and also new and improved medical regimens and practices may allow better matching of diet to insulin through multiple daily injections. However, these factors were not specifically addressed in the current study, and should be explored in future studies.

The results indicate that adolescents with diabetes are not restricted by their illness. They are as physically active, make similar lifestyle choices and have mental health similar to their peers. While previous studies have found elevated levels of disturbed eating behaviours in adolescents with Type 1 diabetes, with estimates of eating disorders ranging from 8 to 30% [4, 24], we found no evidence of disturbed eating. The stronger need for a strict diet to control their eating reported by adolescents with Type 1 diabetes is not surprising given the nature of diabetes. However, the EDS-5 is not a diabetes-specific questionnaire and it is likely that the EDS-5 is insensitive to insulin omission/insulin purging as a means to lose weight. Cigarette smoking has been shown to be an independent risk factor for later medical complications in Type 1 diabetes, and previous reports have shown that around 1 in 4 adolescents smoke on a daily basis [29]. In the current study, only 15% of the adolescents with Type 1 diabetes reported to be smokers, which is similar to both the control group as well as national surveys on current adolescent smoking. We also found no elevated alcohol and drug problems among Type 1 diabetes adolescents, which is important, given that alcohol is associated with impaired ability to detect hypoglycaemic symptoms. One explanation for these non-significant findings may be the corresponding lack of group differences in mental health problems, which previously has been shown to contribute to the initiation of such adverse lifestyle behaviours in diabetes [30]. Of interest is the fact that 15% of adolescents with Type 1 diabetes reported no physical activity at all during the week. Although not significantly higher than in the control group (10%), this figure is disturbingly high, as individuals with Type 1 diabetes face increased risk of cardiovascular diseases, and regular exercise can help reduce these risks. Furthermore, while the rates of mental health issues may not be necessarily elevated in comparison to healthy peers, the implications for untreated mental health disorders in adolescents with chronic illness is significant and even normative levels of psychopathology should be identified given the potential impact on self-care behaviors and health.

Although changes in sleep duration and patterns are well documented in adolescence [31], there is a paucity of studies describing sleep and sleep problems among adolescents with Type 1 diabetes. This is surprising given the relevance of sleep stability in optimizing insulin treatment regimens [7]. In a clinical study of 75 adolescent with Type 1 diabetes, the sleep duration in the Type 1 diabetes group was significantly longer than healthy peers (8.5 hrs vs. 8.0 hrs., respectively). Having Type 1 diabetes was also associated with larger discrepancies between weekends and weekdays [7]. In contrast, the current study found no evidence of altered sleep duration or higher levels of sleep problems.

### Study limitations

There are some methodological limitations of the present study that should be noted. Firstly, the measurement of Type 1 diabetes was based on self-report, and the relatively small number of adolescents with Type 1 diabetes (40 individuals) is a limitation. The adolescent indicated if he/she had diabetes from a checklist, which comprised several chronic illnesses, and the list did not differentiate between Type 1 and Type 2 diabetes. However, in addition the adolescents provided information on all medications they were taking, and all insulins and analogues (ATC–subgroup A10A) were classified as treatments of Type 1 diabetes, and thereby verifying a likely diagnosis. None of the adolescents indicated taking antidiabetic drugs (other than insulin: ATC-subgroup A10B), making it very unlikely that adolescents with Type 2 being were included in the diabetes group. However as adolescents with Type 2 diabetes also may take insulin, we cannot completely rule out the possibility of misclassification. Also, the prevalence of Type 1 diabetes in the current study (0.4%) corresponds well with what has been reported for this age group on a national level in Norway [32]. Still, no clinician verified diagnosis or physiological assessments of blood glucose level or HbA1c were available, and although such assessments are rarely available in large epidemiological studies, the current results should thus be interpreted with caution. Also, the fact that we had no information on use of diabetes technology data limits the ability to investigate whether newer technology (such as new generation of insulin pumps) may be related to mental health functioning. Furthermore, attrition from the study could affect generalizability, with a response rate of about 51% and with adolescents in schools overrepresented. Official data show that in 2012, 92% of all adolescents in Norway aged 16–18 attended high school [33], compared to 98% in the current study. In addition, all 40 adolescents with Type 1 diabetes reported being in high school. As worse health typically increases the risk of being a non-attender in epidemiological studies, it is possible that adolescents with Type 1 diabetes with poor glycaemic control and psychiatric comorbidity might be underrepresented in the current study. Finally, the youth@hordaland study mainly consists of ethnic Norwegians, and the lack of ethic diversity together with the limited age span (16–19 yrs.) may limit the generalizability to other ethnicities and age groups.

## Conclusions

We conclude that there was no evidence of increased psychopathology across a wide range of mental health measures. These findings contrast with earlier studies, and suggest that Type 1 diabetes is not associated with an increased risk of psychosocial problems.
